# Hospital Worthies

**Published:** 1887-05-14

**Authors:** William Marsden

**Affiliations:** Founder of the Royal Free Hospital and Brompton Cancer Hospital


### Hospital Worthies.

### Dr. WILLIAM MARSDEN, Founder of the Royal Free Hospital and Brompton Cancer Hospital.

His Birth and Parentage.

William Marsden was born at Sheffield, August 1796, his father having resided there some years before. The family originally came from Cawthorne, in Yorkshire, where they had been settled for many generations as yeomen or freehold farmers; but at the period of young Marsden's entrance into the world, his grandfather, David Marsden, was residing at Thornhill, a charming rural spot near Dewsbury, farming his own land, as his ancestors had done before him. People in those times rarely moved much about, and in rural districts, year after year, father and son followed in the same occupation. But railways and steam during the last sixty years have changed all this. William Marsden received what in those days was considered a liberal education, and was apprenticed to a wholesale druggist of the town, where he soon became so efficient, that Mr. Hodgkinson, the proprietor of the business, was most anxious he should become a partner, with a view to his ultimately taking the whole concern.

His Early Aspirations.

Young Marsden, however, had a soul above the drug trade, and from an early period longed to quit his provincial friends, and try his fortunes in London. To be a surgeon was the desire of his heart, and a surgeon, too, in London. His holidays, when at school and after, were mostly spent with his grandfather at Thornhill, and it is probable that his first yearnings to follow some scientific career were formed there; for his grandfather, although a farmer, was naturally of a scientific turn, fond of inquiring into the workings of nature, and full of admiration for the works of the Creator. Young Marsden's father was so adverse to his going to London, and so anxious that he should avail himself of Mr. Hodgkinson's offer, that he most positively refused to aid his project in any way; nevertheless, so fully was the young man's mind made up, that, in spite of all opposition, to London he came, before, we believe, he had attained his twentieth year. He arrived in the great city with one £5 note in his pocket, and never wrote home for more. He knew not a soul in London, but we soon find him residing with Mr. Dale, a surgeon in an extensive practice at the top of Holborn Hill, on the same side and some two hundred yards above St. Andrew's Church. Mr. Dale found he had no ordinary youth to deal with, and young Marsden soon became his factotum and favourite assistant.

Enters Bartholomew's.

While here, he entered himself as a student at St. Bartholomew's Hospital, and with his own earnings paid for his medical education. He was a pupil of Abernethy, and a firm believer in his teaching. He remained with Mr. Dale till that gentleman's death, and afterwards carried on the business in conjunction with the nephew of his late employer; but this arrangement does not appear to have been a happy one. Accordingly, Marsden determined to leave, and took a house in Thavies Inn (No. 2, now pulled down for the Holborn Circus). Most of the patients seem to have followed him, for although not yet qualified, we learn that his practice during the first year realised about £1,500. From this time his success became assured. Shortly afterwards he took the M.R.C.S. London, and graduated M.D.

Marsden a Philanthropist.

Thus far we see what young Marsden did for himself; but the great work of his life was not his own advancement, but the amelioration of the conditions on which the destitute wanderer, when overtaken by disease, could obtain admission to our great London hospitals. As we have already related in our story of the Royal Free, Marsden, returning home one midnight in the winter of 1827, found a poor girl actually perishing from want and disease on the steps of St. Andrew's Churchyard. He conveyed her to various London hospitals, but she was refused admission at all, not being provided with the necessary letter of recommendation from a governor, and various other forms about as easy to obtain by a poor sick creature as a feast at a rich man's table. He placed her in a lodging, and two days after she died, unrecognised by a soul. This shocking event, then one of constant occurrence, produced a sad and terrible impression on his mind. He determined that such a disgrace should no longer continue in a Christian country, and to this poor girl's death, witnessed by William Marsden, we owe the foundation, in 1828, of the first free hospital, in the Gray's Inn Road, founded on the principle that disease and destitution shall be the only passport required for admission. This truly great charity has since its foundation relieved 2,000,000 poor and suffering applicants. At a later period Dr. Marsden founded another institution on the same principle, viz., the Cancer Hospital at Brompton. These charities keep upwards of 250 beds always full, and are two of the most complete, successful, and well-managed hospitals in the metropolis. A full account of the foundation of the Royal Free we gave in The Hospital of December 25th, and of the Cancer in that of February 12th. We hear commonly enough of the self-made man who came to London with the proverbial half-crown, and died a rich man, but where shall we find another example of a country lad coming to London utterly unknown, and leaving behind him such monuments to his greatness of soul and goodness of heart as these two noble works?

In 1842 Dr. William Marsden was presented by

A Recognition of his great Work. H.R.H. the late Duke of Cambridge with a magnificent service of plate, consisting of an elegant centre-piece, two wine coolers, and twelve side-dishes, of the value of £800, the money being rapidly subscribed by upwards of 300 persons. The great work which the foundation of these hospitals has accomplished in helping the really poor, in the time of their greatest need, cannot be measured by what has been done by these charities alone, for it has been the means of relaxing the stringent regulations for admission formerly adopted at the other hospitals, and the two institutions have ever been pioneers of good work whenever suddenly called upon. Thus, the Royal Free, at Marsden's suggestion, threw open its door to all sufferers from Asiatic cholera in the visitations of 1832, 1833, 1834, 1848, and 1854, when the other hospitals closed theirs, and we doubt much if any better or more successful plan of treatment that than adopted by him has even yet been discovered. William Marsden worked incessantly for these two charities till his death, which occurred on January 16th, 1867, in his 70th year. He had always a large practice, but, we need hardly say, did not die a rich man. He was beloved by his patients, and those who once consulted him rarely sought the advice of another, and some extraordinary instances of his skill are on record. He published a treatise on the *Treatment of Cholera*, as pursued by him at the Royal Free Hospital, and received for it the special thanks of the Board of Health for the City of London, February 1833. He also translated the late M. Velpeau's work on *Cancer of the Breast*. A full-length portrait, by Pickersgill (exhibited in 1866), hangs in the board-room of the Cancer Hospital, and another, by Illidge (considered the best likeness), may be seen at the Royal Free Hospital.

From the obituary notice (*Medical Times*, January 26th, 1867) we extract the following:—

His Character. "Of the man who accomplished these things, under difficulties which may thus be appreciated, a few words will best convey his individuality. With energy not to be subdued, courage indomitable, self-confidence never shaken, he had the gift of a rare capacity for organising men, and making straight for his object; such was his power of will and intellectual character. Full of worldly wisdom, yet with a deep love of nature; ever kind and hospitable; of simple tastes, playful, sincere, conciliatory, and forgiving,—such were his social and moral qualities. The whole constituting William Marsden will, with what he has achieved in principle and result, pass into history." He was married early in life to Miss Elizabeth Bishop, a young lady whose acquaintance he made on his coach journey to London. This lady was a universal favourite, and contributed in no small degree to the success of the Royal Free Hospital in its earlier days by the geniality of her disposition and the social gatherings held at her house. Dr. Marsden left one son, Dr. Alexander Marsden, who now occupies the position of consulting surgeon to the Royal Free Hospital, and consulting and senior surgeon to the Cancer Hospital, and is one of the trustees of both charities.

